# Neuronal and perineuronal changes of cerebral cortex after exposure to inhaled particulate matter

**DOI:** 10.1038/s41598-019-55956-4

**Published:** 2019-12-19

**Authors:** So Young Kim, Da-hye Lee, Sohyeon Park, Byeong-Gon Kim, An-Soo Jang, Seung Ha Oh, Jun Ho Lee, Myung-Whan Suh, Moo Kyun Park

**Affiliations:** 1 Department of Otorhinolaryngology, CHA University College of Medicine, Seongnam, South Korea; 2 Department of Otorhinolaryngology, Seoul National University College of Medicine, Seoul, South Korea; 3 Division of Allergy and Respiratory Medicine, Department of Internal Medicine, Soonchunhyang University Bucheon Hospital, Bucheon, South Korea; 4 Sensory Organ Research Institute, Seoul National University Medical Research Center, Seoul, South Korea

**Keywords:** Perception, Molecular medicine

## Abstract

The inhalation of particulate matter (PM) increases the perineuronal nets (PNNs) in the cerebral cortex; however, little is known about the related molecular changes. We explored how PM exposure impacted cognitive function and the levels of PNN-related genes. BALB/c mice (6-week-old females, n = 32) were exposed to 1–5-μm diesel-extracted particles (DEPs) (100 µg/m^3^, 5 hours per day, 5 days per week) and categorized into the following four groups: 1) 4-week DEP exposure (n = 8); 2) 4-week control (n = 8); 3) 8-week DEP exposure (n = 8); and 4) 8-week control (n = 8). The Y-maze test and olfactory function test were conducted after 4 and 8 weeks of DEP exposure. The prefrontal cortex, olfactory bulb and temporal cortex were harvested from the animals in each group. The expression of genes related to PNNs (Tenascin C, matrix metalloproteinase [MMP]14, MMP9) and synaptic vesicular transporters of vesicular glutamergic transporter 1 (VGLUT1), VGLUT2, vesicular GABAergic transporter (VGAT) were measured. The temporal cortex was immunostained for neurocan, VGLUT1, and VGAT. The 4-week DEP group had lower total arm entry in the Y-maze test and olfactory sensitivity. These impaired behavioral functions recovered in the 8-week DEP group. Expression of tenascin C and MMP9 were increased in the cerebral cortex in the 8-week DEP group compared with the control group. The levels of VGLUT1, VGLUT2, and VGAT were elevated in the cerebral cortex of the 8-week DEP group compared with the control group. In immunostaining of the temporal cortex, the expression of neurocan, VGLUT1, and GAD67 were increased in the 8-week DEP group compared with the control group. The 4-week DEP inhalation impaired spatial activities and olfactory sensitivities. After 8 weeks of DEP exposure, the PNN components and their proteolytic enzymes and the vesicular transporters increased in the cerebral cortex.

## Introduction

Particulate matter (PM) has been reported to have various adverse health effects, including in the respiratory and cardiovascular systems^1,2^. In addition, a growing body of research has revealed the impacts of PM on the central nervous system. An epidemiologic study demonstrated that short-term exposure to ambient PM (PM_10_ and PM_2.5_) increased the risk of mortality related to cerebrovascular diseases and ischemic stroke^3^. Several animal studies reported cognitive and psychological changes after PM exposure^4,5^. In young mice, 4 weeks of oropharyngeal aspiration of PM diminished spatial learning and memory in association with hippocampal metabolic alterations^4^. Long-term exposure to PM resulted in depressive-like behaviors in mice, with up-regulation of pro-inflammatory cytokines and down-regulation of interleukin-10 and brain-derived neurotrophic factor signalling pathways^5^.

Our previous study demonstrated increased oxidative stress and inflammation in multiple brain regions, including the prefrontal cortex and temporal cortex, after PM inhalation^6^. In addition, the densities of perineuronal nets (PNNs) were elevated in the temporal cortex after 8-week PM inhalation. However, the underlying molecular changes of PNNs and synaptic transporters after PM inhalation remain unknown. PNNs consist of large molecules, including proteoglycans, tenascin and linking proteins, that cover the neuron’s body and dendrites^7^. PNNs are known to be very important in synaptic function and neuroplasticity^7^, and stabilize synapses^8^. Breakdown of nets produces unwanted sprouting of synapses and changes in the balance of excitatory and inhibitory neuronal transmission. For instance, parallel plastic changes of excitatory and inhibitory synaptic transporters of vesicular glutamergic transporter 1 (VGLUT1), VGLUT2 and vesicular GABAergic transporter (VGAT), as well as changes in PNN densities, were observed during vestibular compensation^9^.

We postulated that PM inhalation impaired spatial cognitive function and olfactory sensitivities. These behavioral changes might accompany the dynamic changes of PNNs encompassing degradation as well as synthesis. Furthermore, these changes in PNNs might be linked to both excitatory and inhibitory changes in synaptic transporter levels. To test this hypothesis, spatial learning memory function and olfactory function tests were performed after PM inhalation. In addition, components of PNNs, their proteolytic enzymes, and excitatory and inhibitory vesicular transporters were evaluated for longitudinal changes after PM inhalation. No prior study has described synaptic vesicular transporters and molecular changes of PNNs after PM exposure.

## Results

### Effect of DEP exposure on olfactory function and working memory as revealed by the olfactory and Y-maze tests

**Figure 1:**
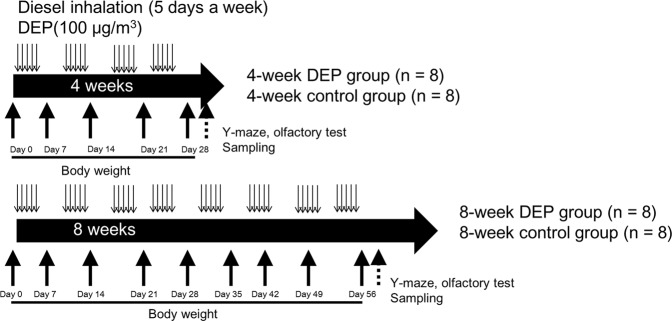
Schedule of diesel-extracted particle (DEP) exposure. Mice inhaled DEPs for 4 or 8 weeks (n = 8/group).

**Figure 2:**
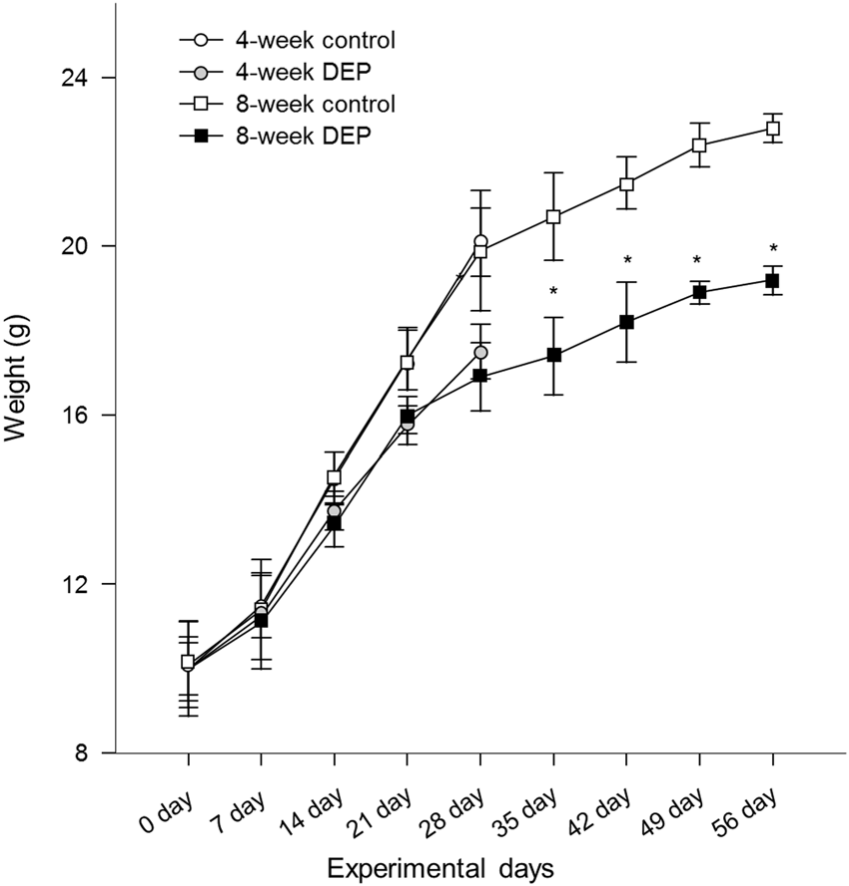
Changes in body weight by days of DEP exposure (n = 8/group, *P < 0.05 on Mann–Whitney U-testing of the control vs. the DEP group).

**Figure 3:**
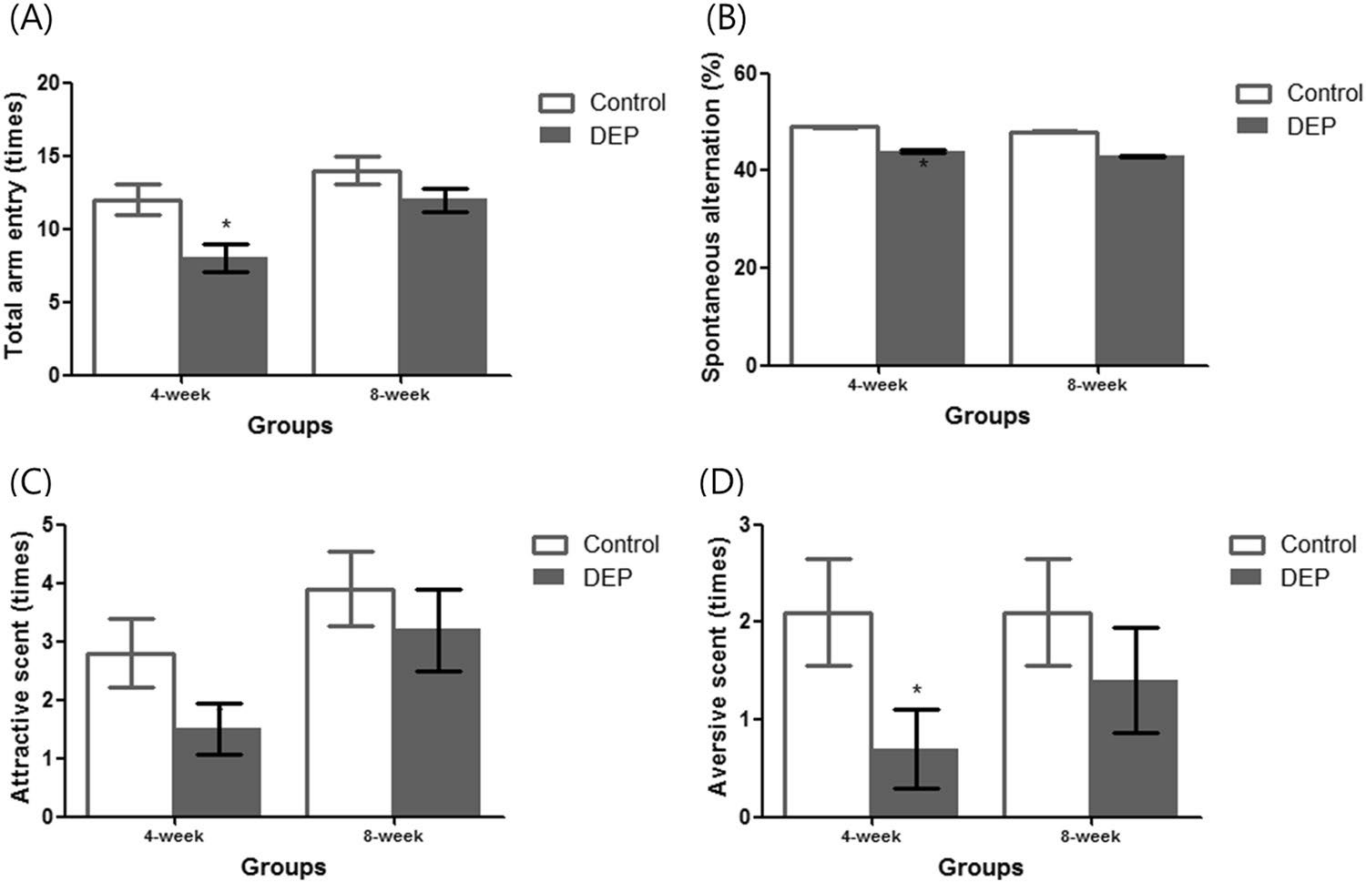
Effects of DEP exposure on the Y-maze test results. (**A**) The 4-week DEP group exhibited fewer total arm entries than the 4-week control group (**B**). The spontaneous alternation percentages were comparable between the two groups (**C**). The number of attractive scent sniffs was lower in the 4-week DEP group than the 4-week control group (**D**). The number of aversive scent sniffs was lower in the 4-week DEP group than the 4-week control group (n = 8 for each group, *P < 0.05 on Mann–Whitney U-testing of the control vs. the DEP groups).

Body weights, and the Y-maze and olfactory test results, were compared between the control and the 4-/8-week DEP groups (Fig. 1). Body weight did not change from commencement of the experiment to 4 weeks later. However, body weight was significantly lower in the 8-week DEP group than in the 8-week control group (Fig. 2). Four weeks of DEP exposure reduced the number of total arm entries compared to that of the control group (Fig. 3A and Supplemental Table S1). However, the numbers of total arm entries of the 8-week DEP and control groups were comparable. The spontaneous alternation percentages were similar among the four groups (Fig. 3B). On olfactory testing, the 4-week DEP group exhibited fewer sniffs of both attractive and aversive scents than the control group (Fig. 3C,D).

### Changes in neurocan and tenascin C levels

**Figure 4:**
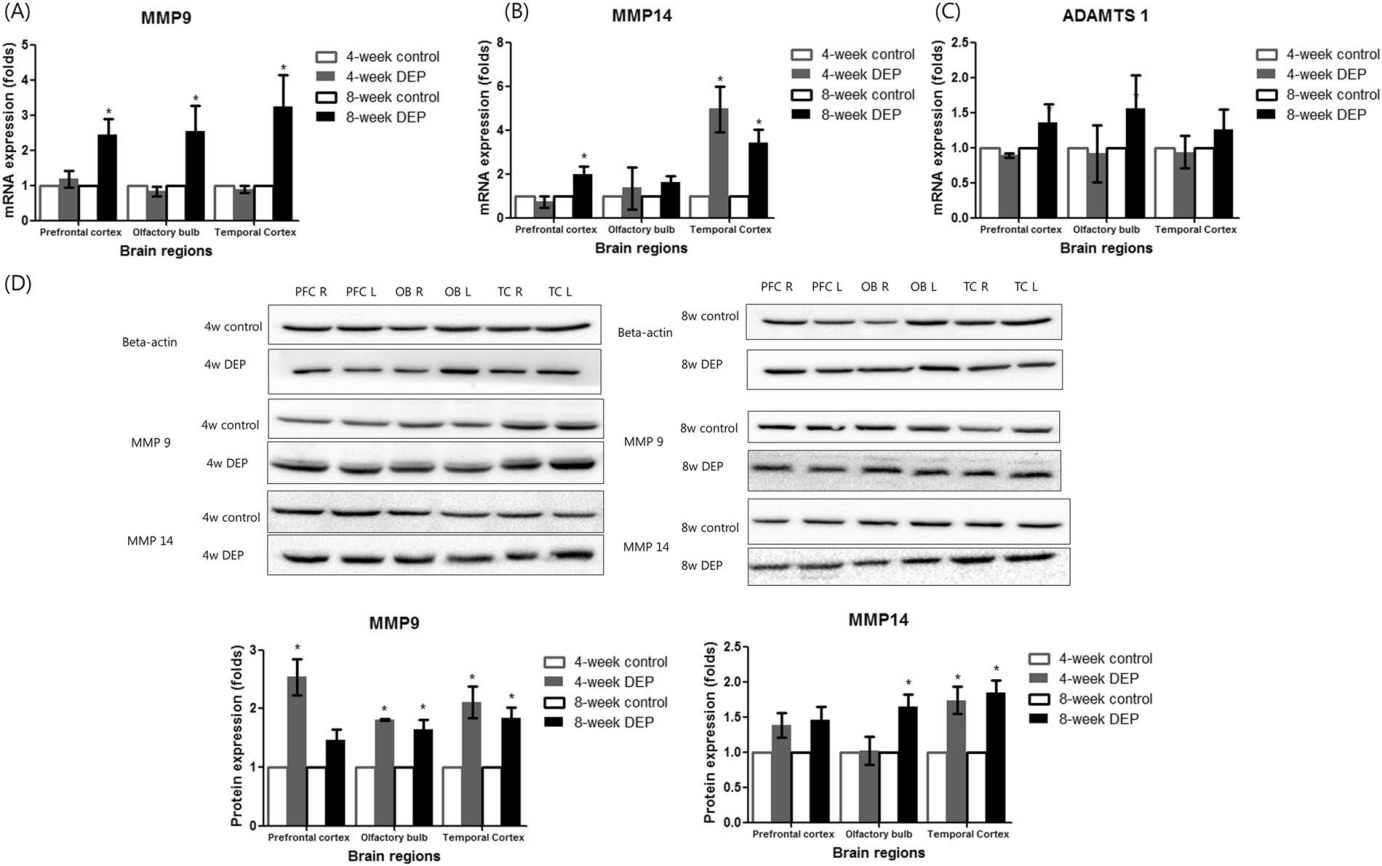
Changes in the levels of mRNAs encoding matrix metalloproteinase (MMP) 9 (**A**), MMP 14 (**B**), and a disintegrin and a metalloproteinase featuring the thrombospondin motif ADAMTS 1 (**C**) in the frontal cortex, olfactory bulb, and temporal cortex after 4 or 8 weeks of DEP inhalation, compared to the control group. (**D**) The MMP 9 and MMP 14 protein expression levels after 4 weeks of DEP inhalation compared to those of controls. The 4- and 8-week data were derived using different gels. n = 5/group, *P < 0.05 on Mann–Whitney U- testing (control vs. DEP groups) PFC: prefrontal cortex, OB: olfactory bulb, TC: temporal cortex.

The expression levels of the lectican-cleaving protease MMP 9 were higher in the prefrontal cortex, olfactory bulb, and temporal cortex of the 8-week DEP group than in those tissues of the control group (Fig. 4A and Supplemental Table S2). The level of mRNA encoding MMP14 was higher in the temporal cortex of the 4-week DEP group than the control group (Fig. 4B). The levels of MMP9 and MMP14 proteins increased in the DEP groups (Fig. 4D and Supplemental Fig. 1). The ADAMTS1 level tended to increase in the prefrontal cortex, olfactory bulb, and temporal cortex of the 8-week DEP group (compared to controls), but statistical significance was not attained (Fig. 4C).

**Figure 5:**
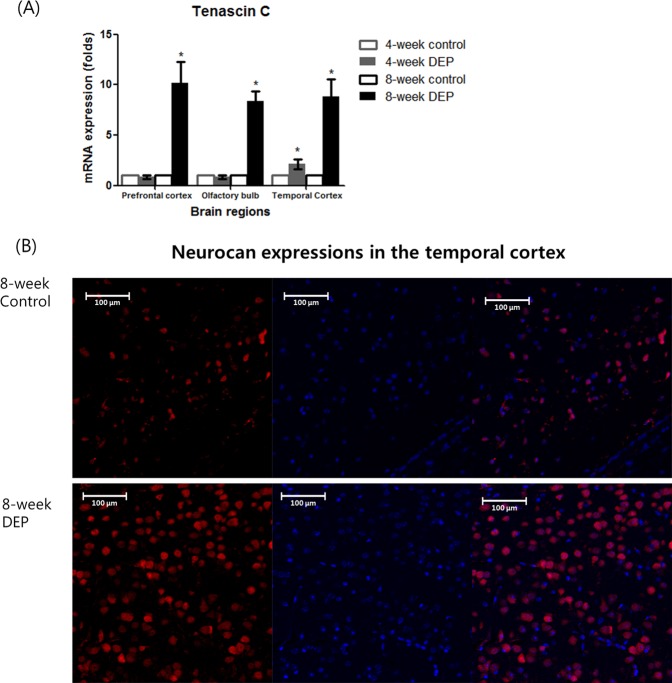
(**A**) Changes in the levels of mRNA encoding Tenascin C in the prefrontal cortex, olfactory bulb, and temporal cortex after 4 or 8 weeks of DEP inhalation compared to the control group (n = 5/ group, *P < 0.05 on Mann–Whitney U-testing (control vs. DEP groups) (**B**). Changes in the numbers of neurocan (red)-positive cells in the temporal cortex of the 8-week DEP group.

The tenascin C levels were higher in the prefrontal cortex, olfactory bulb, and temporal cortex of the 8-week DEP group than in those tissues of the control group (Fig. 5A and Supplemental Table S3). The levels in the 4-week DEP group were comparable to those of controls. In the immunofluorescence assay, the neurocan level in the temporal cortex was higher (as evidenced by greater numbers of immunopositive cells) in the 8-week DEP group compared to the control group (Fig. 5B).

### Changes in the levels of excitatory and inhibitory vesicular transporters

**Figure 6:**
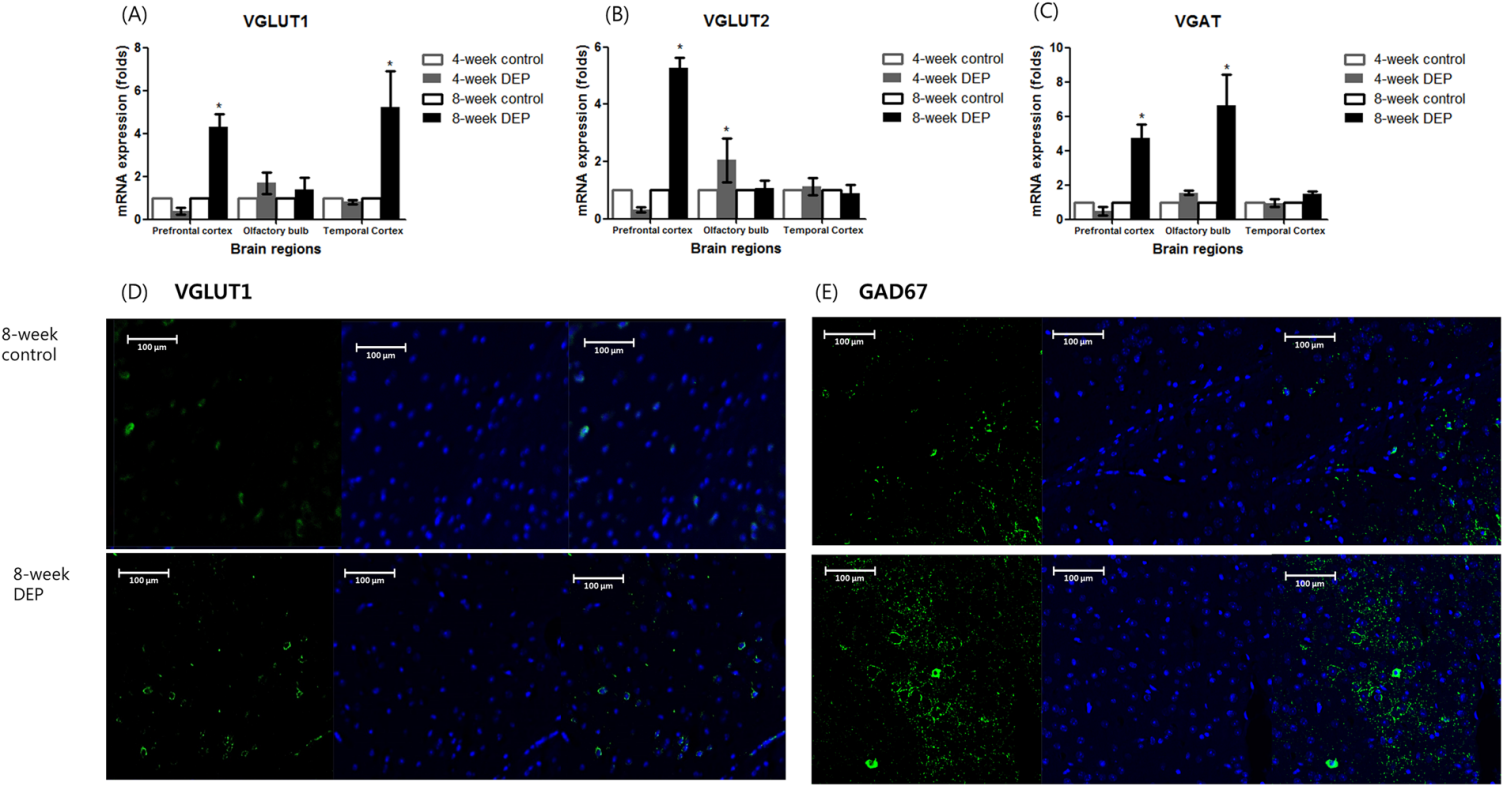
Changes in the levels of mRNAs encoding the vesicular glutamergic transporter (VGLUT)1 (**A**), VGLUT2 (**B**), and the vesicular GABAergic transporter (VGAT) (**C**) in the prefrontal cortex, olfactory bulb, and temporal cortex after 4 or 8 weeks of DEP inhalation, compared to those of the control group (n = 5/group; *P < 0.05 on Mann–Whitney U-testing comparing the control and DEP groups). Changes in the numbers of vesicular glutamergic transporters (VGLUT)1 (green) (**D**); glutamate acid decarboxylase (GAD)67 molecules (green) (**E**); and neurocan (red)-positive cells in the temporal cortices of the 8-week DEP-exposed group. The numbers of VGLUT1- and GAD67-postive cells were higher in the 8-week DEP group than the control group (blue: DAPI).

The VGLUT1 levels were higher in the prefrontal and temporal cortices of the 8-week DEP group compared to the control group (Fig. 6A and Supplemental Table S3). The VGLUT1 expression levels were comparable between the 4-week DEP and control groups. The olfactory bulb VGLUT1 expression level did not differ between the 4- and 8-week DEP groups and the control group. VGLUT2 levels were elevated in the prefrontal cortex of the 8-week DEP group and in the olfactory bulb of the 4-week DEP group (Fig. 6B). All groups exhibited comparable VGLUT2 levels in the temporal cortex. VGAT levels were increased in the prefrontal cortex and olfactory bulb of the 8-week DEP group compared to controls (Fig. 6C). Temporal cortex VGAT levels were similar in all groups.

In the immunofluorescence assay, the VGLUT1 level in the temporal cortex was elevated (as evidenced by greater numbers of immunopositive cells) in the 8-week-DEP group compared to the control group (Fig. 6D). The extent of GAD67-immunopositivity was higher in the 8-week DEP group than in the control group (Fig. 6E).

## Discussion

### Principal findings of the present study

Four weeks of DEP exposure reduced spatial activity and olfactory sensitivity compared to those of the control group. Although spatial recognition memory was not definitively impaired, spatial mobilization was decreased in the 4-week DEP group. Such immobility is suggestive of depressive behavior^10–12^. These changes became normalized after 8 weeks of DEP exposure. However, weight loss was more significant in the 8- then 4-week DEP group (compared to the control group). In molecular terms, the PNN levels, as in our previous study^6^, and the levels of neurocan and tenascin C (in our current study) were increased after 8 weeks of DEP exposure. Moreover, the temporal cortex levels of the lectican-cleaving proteases MMP9 and MMP14 increased after 8 weeks of DEP exposure. Thus, both PNN degradation and synthesis may increase after 8 weeks of DEP exposure. The increases in the levels of excitatory vesicular transporters and MMPs were accompanied by increases in the excitatory vesicular transporters levels. The levels of excitatory and inhibitory vesicular transporters increased after 8 weeks of DEP inhalation. The levels of excitatory vesicular transporters were elevated in the cerebral cortex. The levels of inhibitory vesicular transporters increased in the prefrontal cortex and olfactory bulb. Such molecular changes may attenuate the depressive and olfactory behavioral changes^13–15^.

### Increases in PNN component and MMP9/14 levels after 4-week DEP exposure

The levels of PNN components and related molecules (neurocan, tenascin C, MMP9, and MMP14) increased after 4-week DEP inhalation, consistent with our previous finding that PNN density increased in an 8-week DEP group^6^.

MMP9 and MMP14 levels were elevated in the temporal cortex. The protein level of MMP9 were increased in both 4-week and 8-week DEP exposure groups, although the mRNA level of MMP9 showed significant increase only in the 8-week DEP exposure group in the present study. These discrepancies could be caused by the potential activation and secretion of MMP9 in protein levels, which preceded the up-regulation of MMP9 transcription. MMPs are secretory endopeptidases regulating PNN formation by cleaving PNN components. MMP9 is a secreted inducible enzyme that is activated both by free radicals and other enzymes or factors^16^. For instance, sorting nexin (SNX) 10 promoted the activation and secretion of MMP9, and the inhibition of SNX 10 reduced the MMP9 activities although the protein levels of MMP9 were increased^17^. In addition, the protein level of MMP9 in the western blotting may include both pro- and active- MMP9. To discriminate the active-MMP9, the secreted MMP9 measurement using enzyme linked immunosorbent assay or zymography assay will be needed^18^. A previous study using MMP9-deficient mice reported that MMP9 played a crucial role in PNN remodeling^19^. Extracellular matrix components, including CSPGs and other surface molecules, are substrates of MMP14^20,21^. MMP14 expression is upregulated in patients with neurodegenerative diseases such as Alzheimer’s disease, multiple sclerosis and stroke, as well as neuroinflammatory diseases^22^. Therefore, the observed increases in MMP9 and MMP14 levels suggest possible neurological effects of 4-week DEP exposure; both synthetic and degradative changes in PNNs were apparent.

Temporal cortex neurocan and tenascin C levels increased since 4 weeks of DEP exposure. Neurocan is a chondroitin sulfate proteoglycan (CSPG), thus composed of PNNs. Tenascin C is an ECM glycoprotein that mediates changes in neuronal plasticity via several mechanisms^19,23^. The protein interacts with several CSPGs of PNNs^19^. In the adult central nervous system, tenascin C is expressed in regions of active neurogenesis and areas exhibiting neuronal plasticity^19,23,24^. The observed rise in tenascin C levels suggests that PNN synthesis is activated after 4-week DEP exposure. Such exposure may affect synaptic functions by changing PNN levels. Neurocan modulates the level of PNN structural constituents, actively remodels the synapses of GABAergic interneurons by binding to the immunoglobulin-class cell-adhesion molecule NCAM, and competitively inhibits the tyrosine kinase EphA3^25^. Increased levels of PNN components may upregulate the presynaptic glutamergic transporter. A recent, synaptic fluorescence imaging study showed that increased PNN levels in the cerebral cortex were accompanied by VGLUT2 upregulation^26^. However, VGAT did not modulate inhibitory synapse formation in interneurons, or the PNN levels thereof^27^. This may explain why no definitive change in VGAT expression level in the temporal cortex was evident in the current study.

### Increases in the levels of excitatory and inhibitory vesicular transporters after 8-week DEP exposure

The levels of both excitatory and inhibitory presynaptic transporters increased after 8 weeks of DEP exposure. VGLUT1 expression increased to a greater extent than VGLUT2 expression after such exposure. VLUGT1 is the predominant moiety of the corticocortical and corticothalamic projection systems^28^. VGLUT2 is the principal vesicular glutamate transporter of the subcortical and thalamocortical circuits^28^. Thus, the results suggest that DEP-induced changes occurred principally at the level of the cerebral cortex. Similarly, several previous studies reported elevations in the synaptic glutamate level and GABA transport after particle exposure^29,30^. One *in vitro* study found that PM-exposed microglia exhibited increased levels of extracellular glutamate, correlating with neuronal viability^30^. In addition, presynaptic glutamate and GABA accumulated in a dose-dependent manner following exposure to carbon dots^29^. Another study reported that the levels of mRNAs encoding the GABAB1 and GABAB2 receptors rose in the prefrontal cortex of rats exposed to cigarette smoke^31^. However, changes in synaptic transporter levels did not become significant before 8 weeks of DEP exposure in the present study, whereas depressive behavior and olfactory desensitization were apparent after 4 weeks of such exposure. Although the levels of vesicular synaptic transporters did not change, the observed neuroinflammatory alterations may have contributed to the behavioral changes evident after 4 weeks of DEP exposure^6^. In addition, the moderately high concentration of DEP (100 μg/m^3^) used in the present study may be too low to trigger early changes in the levels of vesicular synaptic transporters; exposure to DEP for 8 weeks might be necessary to increase transporter levels. In the current study, cerebral cortex VGLUT1 and VGLUT2 levels increased after 8 weeks of DEP exposure. Such alterations in vesicular neurotransmission levels may have mediated recovery from depressive behavior triggered by such exposure. Previous studies found that cerebral cortical VGLUT1 and VGLUT2 levels increased as depression became relieved^13,14^. Similarly, VGAT expression was elevated in the prefrontal cortex and olfactory bulb after 8 weeks of DEP exposure. An increase in vesicular GABA transmission may have aided recovery of olfactory bulb olfaction^15^. However, weight loss increased with the duration of DEP exposure; such exposure may have been accompanied by stress-induced depressive behavior^32^.

In conclusion, 4-week DEP inhalation induced depressive behavior and decreased olfactory sensitivity, but recovery was evident after longer-term DEP exposure. Although the behavioral changes became normalized, MMP9 and MMP14 expression levels increased after 8-week DEP inhalation, perhaps explaining the prolonged degenerative and inflammatory changes noted in the cerebral cortex. The recovery of behavioral changes were related to increases in the levels of excitatory and inhibitory vesicular transporters and extracellular matrix components in the cerebral cortex.

## Materials and Methods

### Animal experiments

The study was approved by the Institutional Animal Care and Use Committee of Soonchunhyang University Medical School (SCHBC-Animal-2014-013). All methods adhered to the guidelines and regulations of the Institutional Animal Care and Use Committee of Soonchunhyang University Medical School. BALB/c mice (6-week-old females, n = 32) were divided into four groups (n = 8 for each group): (1) 4-week diesel-extracted particle (DEP) exposure, (2) 4-week control, (3) 8-week DEP exposure and (4) 8-week control. Mice were raised under standard conditions and exposed to DEP (NIST SRM 2975, Sigma-Aldrich) (5–10 μm in diameter) as described in a previous study^6,33^. DEP was autoclaved and coated with BAS to prevent aggregation. DEP was suspended in serum-free medium, nebulized, and delivered at 0.25 mL/min using a MEDI-PUMP (model #1125; Thomas Corporation, IL, USA). The 4- and 8-week DEP groups were exposed to 100 μg/m^3^ DEP for 5 h per day on 5 days per week for 4 and 8 weeks respectively (Fig. 1). The control mice were exposed to saline solution for periods identical to those of the experimental groups. Fresh tissues from the prefrontal cortex, olfactory bulb and temporal cortex (n = 5 for each exposure group), as well as whole brain tissue for immunostaining (n = 3 for each exposure group), were harvested at the end of the experimental period (4 or 8 weeks). To minimize circadian effects, all tissue harvest experiments were performed at a consistent time, around 4 p.m.

### Behavioral tests

Body weights were measured before (day 0) and every 7 days (i.e. days 7, 14, 21, 28, 35, 42, 49 and 56) after DEP exposures in all mice. The Y-maze test for spatial working memory and olfactory sensitivity test were conducted 4 weeks and 8 weeks after DEP exposure in the 4-week DEP/control group and 8-week DEP/control group, respectively.

### Spatial working memory in the Y-maze

Animals were acclimated to the testing area for 30 min prior to the test. A black plastic Y-maze was used to differentiate the color of the maze and the mouse. The Y-maze consisted of three identical arms (3 cm wide, 32.5 cm long, 15 cm high, 120° apart) placed in the testing room. Each arm was named A, B and C (clockwise from the top). Individual mice were placed at the end of one of the arms facing the center and allowed to explore the maze freely for 5 min. The maze was cleaned after each session. The tests were video recorded and analyzed manually by two blinded investigators. Entries were counted when the animals passed through the midpoint of the arm. Entries into new arms were considered successful alternations, as opposed to entering the two previously visited arms. Alternation percentage was calculated as shown below.

$$ \% \,{\rm{Alternation}}=({\rm{Number}}\,{\rm{of}}\,{\rm{successful}}\,{\rm{alternations}}/[{\rm{Total}}\,{\rm{arm}}\,{\rm{entries}}-2]\,)\,\ast \,100$$

### Olfactory sensitivity test

Animals were habituated to the testing area for 30 min prior to the test. During the test and resting phases, animals were placed in a clean, empty cage; cages were cleaned after each test. Animals were introduced to distilled water as a control scent, 10% peanut butter solution as an attractive scent and 10% trichloroacetic acid (TCA) solution as an aversive scent. A 2 × 2 in square piece of filter paper was impregnated with each scent and placed in one side of the cage. Animals were exposed to each scent for 3 min and all sessions were video recorded. All animals went through the resting phase in another clean, empty cage for 6 min between sessions to neutralize the scent they were exposed to previously. The total number of sniffs were analyzed after the experiment.

### Analysis of mRNA expression

Fresh olfactory bulb, prefrontal cortex, and temporal cortex tissues were micro-punched and rapidly frozen at −20 °C on dry ice. Using the TRI Reagent® (Sigma-Aldrich), total RNA was extracted from mouse brain tissues according to the manufacturer’s instructions. Reverse transcription was performed using TOPscript^TM^ RT DryMIX (dT18 plus; Enzynomics Co. Ltd., Daejeon, South Korea) as in our previous study^6^. Table 1 lists the forward and reverse oligonucleotides used for PCR amplification of the markers of the PNNs (tenascin C, matrix metalloproteinase [MMP]14, MMP9 and a disintegrin and metalloproteinase with thrombospondin motifs 1 [ADAMTS1]) and the vesicular transporters of VGLUT1, VGLUT2, and VGAT. All mRNA levels were expressed as percentages of the mRNA level encoding glyceraldehyde 3-phosphate dehydrogenase (GAPDH). All RT-PCR reactions were replicated three times for each gene from five mice in each group.

**Table 1:** The primers used for quantitative reverse transcriptase polymerase chain reaction.

Gene	Primer sequence (forward)	Primer sequence (reverse)	Annealing temperature (°C)	Product size (bp)
VGLUT1	5′-TGCTGAGTCCGCAGCAGGTG-3′	5′-GCTGGCAGGCTCTGGGGAAG-3′	60	169
VGLUT2	5′-CAGACCTGCCTTACGACTATGG-3′	5′-CTCGGTGGCGTTGAGATTGTT-3′	60	113
VGAT	5′-CATTGCTCAAGTGTCTGAAGC-3′	5′-CATGGCCACAACAACTGACG-3′	60	100
MMP9	5′-AAGCCTCTAGAGACCACCCC-3′	5′-CTTCTGACCAACCACAGGCT-3′	60	230
MMP14	5′-GGACTTTGCCTCCTCCGA-3′	5′-GACCATCTTCTGCTCAGCCC-3′	60	177
TenascinC	5′-GCTCTCCTATGGCATCAAGG-3′	5′-TCATGTGTGAGGTCGATGGT-3′	55	60
ADAMTS1	5′-GCACT CAAGGCGTAGGAC-3′	5′-AAGCATGGT TTCCACATGCG-3′	62	89

### Western blotting

Proteins were extracted from brain tissue and subjected to Western blotting as previously described^6^. Approximately 20 μg of protein was separated by 12% sodium dodecyl sulphate-polyacrylamide gel electrophoresis (SDS-PAGE) and transferred to polyvinylidene difluoride (PVDF) membranes (Merck Millipore, Burlington, MA, USA). The membranes were soaked in blocking buffer (5% non-fat dry milk in Tris-buffered saline containing Tween-20 [TBS-T]) for 1 h at room temperature. They were then incubated with specific primary antibodies: anti-MMP9 (rabbit polyclonal; Abcam, Cambridge, UK), anti-MMP14 (rabbit monoclonal; Abcam) and β-actin (D6A8, rabbit mAb; Cell Signaling Technology). Immunoreactive proteins were detected with a horseradish peroxidase (HRP)-coupled secondary antibody (anti-rabbit IgG, HRP-linked antibody; Cell Signaling Technology) and visualized using an enhanced chemiluminescence (ECL) kit (Bio-Rad). The protein bands were quantitated by densitometry using ImageJ gel analysis software (National Institutes of Health, Bethesda, MD, USA).

### Immunofluorescence study

Whole brains were immersion-fixed in 4% (v/v) paraformaldehyde. The specimens were dehydrated and embedded in paraffin with optimal cutting temperature solution. For histological examination, 10-µm sections of embedded tissue were cut on a rotary microtome and mounted on glass slides. Each slide was dipped in xylene for 10 min to remove paraffin, followed by sequential washes with 100, 75 and 50% ethanol for 5 min at each concentration. Immunofluorescence was progressed by free-floating sections three times for 5 min each in PBS. After a set of three 5-min washes in PBS, the sections were placed in 10% goat or donkey blocking serum (Vector Labs, Burlingame, CA, USA) for 1 hour at room temperature. Free-floating slices were then incubated overnight at 4 °C on a shaking table with primary antibodies (rabbit anti-neurocan, rabbit anti-VGLUT1, and mouse anti-GAD67). The following day, after three 10-min washes in PBS, sections were incubated for 2 h in secondary antibodies (goat anti-rabbit alexa 488, goat anti-mouse alexa 488 and goat anti-guinea pig alexa 555) at room temperature. After another PBS wash, DAPI solution was dropped on each slide and left for 5 min. The tissue was washed three additional times in PBS for 10 min per wash and mounted onto slides. After drying, the slides were cover-slipped. The temporal cortex (A1) was localized by reference to a mouse brain atlas^34^. Six A1 areas from each group were analyzed in a 777 × 777 µm area. Images were photographed using a TCS SP5II confocal microscope (Leica, Wetzlar, Germany).

### Statistical analysis

Statistical analysis was performed using the Mann–Whitney U test after testing for normality with the Kolmogorov–Smirnov and Shapiro–Wilk tests. Values are expressed as means with standard error of the mean. SPSS software (ver. 21.0; IBM Corp., Armonk, NY, USA) was used for the analyses. A *P*-level ≤ 0.05 was considered to reflect statistical significance.

## Supplementary information

Supplement Table
