# Supplementary material for: Neuronal and perineuronal changes of cerebral cortex after exposure to inhaled particulate matter
Source: Sci Rep. 2019 Dec 19;9:19421. doi: 10.1038/s41598-019-55956-4 (PMC6923377; doi:10.1038/s41598-019-55956-4)

**Neuronal and perineuronal changes of cerebral cortex after exposure to inhaled particulate matter**

So Young Kim^1^, Da-hye Lee^1^, Sohyeon Park^2^, Byeong-Gon Kim^3^, An-Soo Jang^3^, Seung Ha Oh^2,4^, Jun Ho Lee^2,4^, Myung-Whan Suh^2,4^, Moo Kyun Park^2,4*^

^1^Department of Otorhinolaryngology, CHA University College of Medicine, ^2^Department of Otorhinolaryngology, Seoul National University College of Medicine, ^3^Division of Allergy and Respiratory Medicine, Department of Internal Medicine, Soonchunhyang University Bucheon Hospital, ^4^Sensory Organ Research Institute, Seoul National University Medical Research Center. Seoul, South Korea

Running title: Effects of inhaled particulate matter on the cerebral cortex

***Correspondence**: parkaseptic@daum.net

**Conflicts of interest**: none

Supplement Table S1 The comparisons of Y-maze tests and olfactory tests between control and diesel-extracted particles (DEP) groups

|  | | Control | | DEP | | P-value | |
| --- | --- | --- | --- | --- | --- | --- | --- |
| Total arm entry (times) | |  | |  | |  |  |
| 4-week | | 11.86 (1.03) | | 7.83 (0.96) | | 0.05* | |
| 8-week | | 14.36 (0.98) | | 11.57 (0.81) | | 0.09 | |
| Spontaneous alternation (%) | |  | |  |  |  |  |
| 4-week | | 48.83 (0.24) | | 44.39 (0.27) | | 0.67 | |
| 8-week | | 47.66 (0.20) | | 43.29 (0.22) | | 0.59 | |
| Attractive scent (times) | |  | |  | |  |  |
| 4-week | | 2.75 (0.58) | | 1.50 (0.44) | | 0.22 | |
| 8-week | | 3.93 (0.65) | | 3.21 (0.70) | | 0.41 | |
| Aversive scent (times) | |  | |  | |  |  |
| 4-week | | 2.08 (0.55) | | 0.70 (0.40) | | 0.01* | |
| 8-week | | 2.07 (0.55) | | 1.36 (0.54) | | 0.21 | |

*P ≤ 0.05 (Mann–Whitney U test between control vs. DEP groups)

Supplement Table S2 The comparisons of protein expression levels of matrix metalloproteinase (MMP) 9 and MMP14 between control and diesel-extracted particles (DEP) groups

|  | | DEP 4-week | | P-value | | DEP 8-week | | P-value | |
| --- | --- | --- | --- | --- | --- | --- | --- | --- | --- |
| MMP9 | |  | |  | |  | |  | |
| Prefrontal cortex | | 2.54 (0.30) | | 0.050* | | 1.46 (0.19) | | 0.187 | |
| Olfactory bulb | | 1.81 (0.02) | | 0.046* | | 1.64 (0.17) | | 0.050* | |
| Temporal Cortex | | 2.11 (0.27) | | 0.043* | | 1.84 (0.17) | | 0.050* | |
| MMP14 | |  | |  | |  | |  | |
| Prefrontal cortex | | 1.38 (0.17) | | 0.870 | | 1.46 (0.19) | | 0.187 | |
| Olfactory bulb | | 1.02 (0.20) | | 0.200 | | 1.64 (0.17) | | 0.050* | |
| Temporal Cortex | | 1.74 (0.19) | | 0.050* | | 1.84 (0.17) | | 0.050* | |

*P ≤ 0.05 (Mann–Whitney U test between control vs. DEP groups)

Supplement Table S3 The comparisons of mRNA expression levels between control and diesel-extracted particles (DEP) groups

|  | | DEP 4-week | | P-value | | DEP 8-week | | P-value | |
| --- | --- | --- | --- | --- | --- | --- | --- | --- | --- |
| VGLUT1 | |  | |  | |  | |  | |
| Prefrontal cortex | | 0.38 (0.16) | | 0.063 | | 4.29 (0.59) | | 0.038* | |
| Olfactory bulb | | 1.69 (0.51) | | 0.053 | | 1.38 (0.56) | | 0.203 | |
| Temporal Cortex | | 0.82 (0.11) | | 0.276 | | 5.21 (1.70) | | 0.031* | |
| VGLUT2 | |  | |  | |  | |  | |
| Prefrontal cortex | | 0.32 (0.09) | | 0.054 | | 5.26 (0.37) | | 0.021* | |
| Olfactory bulb | | 2.05 (0.75) | | 0.050* | | 1.06 (0.29) | | 0.857 | |
| Temporal Cortex | | 1.14 (0.30) | | 0.698 | | 0.88 (0.32) | | 0.739 | |
| VGAT | |  | |  | |  | |  | |
| Prefrontal cortex | | 0.48 (0.24) | | 0.186 | | 4.73 (0.78) | | 0.037* | |
| Olfactory bulb | | 1.54 (0.13) | | 0.185 | | 6.64 (1.81) | | 0.020* | |
| Temporal Cortex | | 0.94 (0.22) | | 0.856 | | 1.47 (0.14) | | 0.187 | |
| TenascinC | |  | |  | |  | |  | |
| Prefrontal cortex | | 0.83 (0.19) | | 0.385 | | 10.15 (2.14) | | 0.001* | |
| Olfactory bulb | | 0.85 (0.17) | | 0.651 | | 8.38 (0.99) | | 0.014* | |
| Temporal Cortex | | 2.10 (0.48) | | 0.047* | | 8.82 (1.76) | | 0.015* | |
| MMP9 | |  | |  | |  | |  | |
| Prefrontal cortex | | 1.18 (0.24) | | 0.348 | | 2.43 (0.46) | | 0.045* | |
| Olfactory bulb | | 0.83 (0.15) | | 0.532 | | 2.54 (0.72) | | 0.043* | |
| Temporal Cortex | | 0.90 (0.11) | | 0.739 | | 3.25 (0.89) | | 0.040* | |
| MMP14 | |  | |  | |  | |  | |
| Prefrontal cortex | | 0.74 (0.27) | | 0.211 | | 1.97 (0.39) | | 0.050* | |
| Olfactory bulb | | 1.36 (0.96) | | 0.210 | | 1.64 (0.28) | | 0.060 | |
| Temporal Cortex | | 4.96 (1.04) | | 0.036* | | 3.42 (0.61) | | 0.040* | |
| ADAMTS1 | |  | |  | |  | |  | |
| Prefrontal cortex | | 0.89 (0.03) | | 0.735 | | 1.35 (0.27) | | 0.212 | |
| Olfactory bulb | | 0.92 (0.41) | | 0.749 | | 1.56 (0.48) | | 0.210 | |
| Temporal Cortex | | 0.94 (0.23) | | 0.754 | | 1.25 (0.29) | | 0.187 | |

*P ≤ 0.05 (Mann–Whitney U test between control vs. DEP groups)

Supplement Fig. 1 The full-length blots for the MMP9 and MMP 14 in figure 4 (d). Because the full-length blot for figure 4 (d) was discarded, the repeated experiments were performed for the identical conditions.


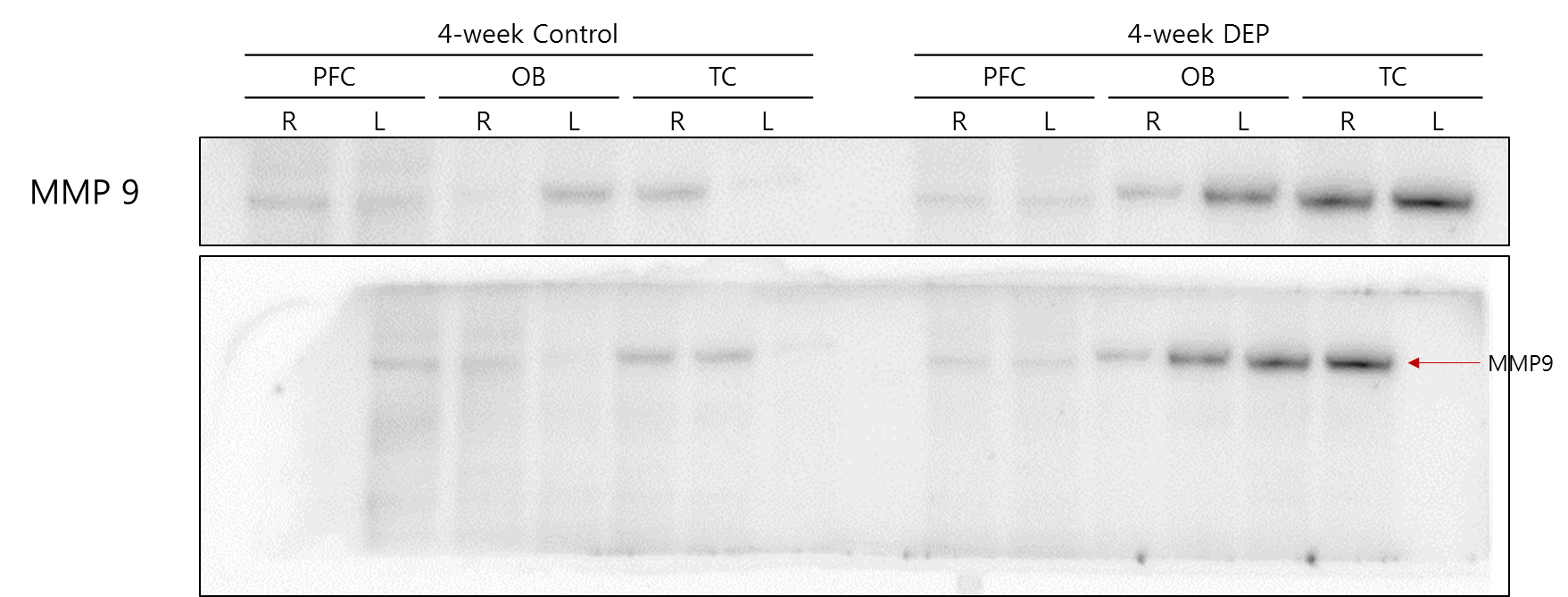


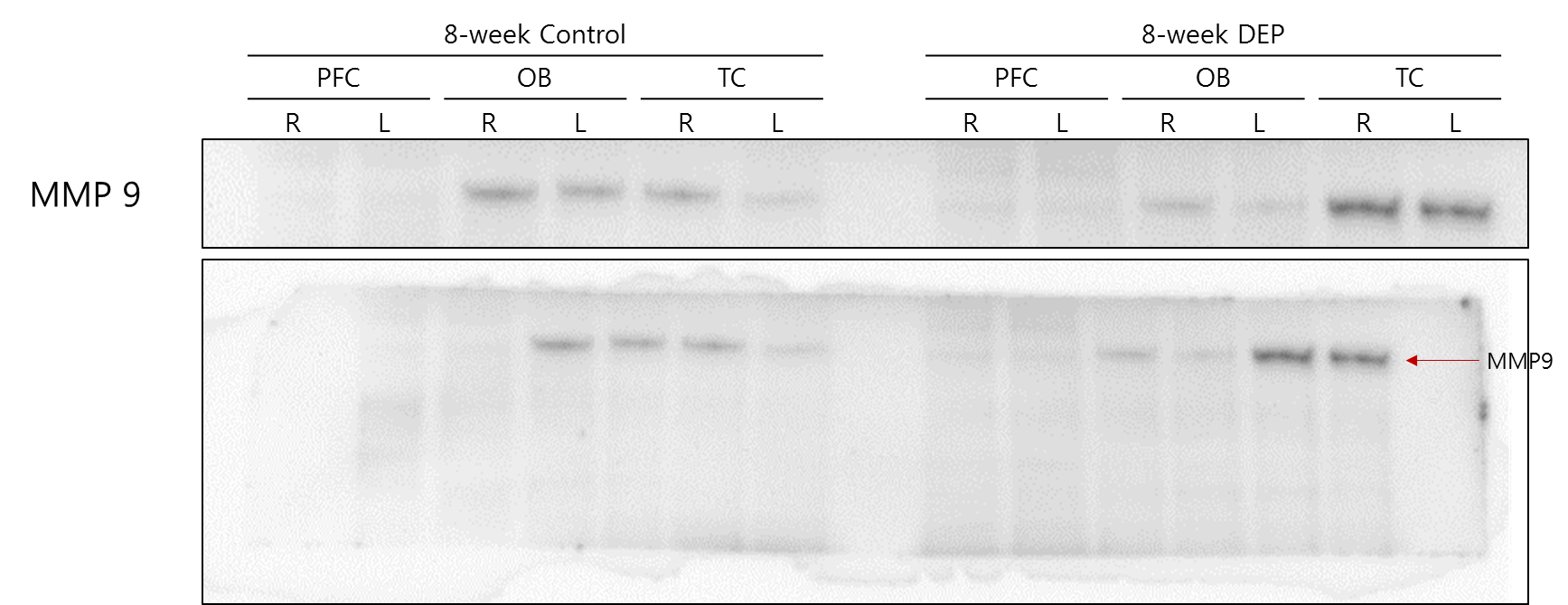


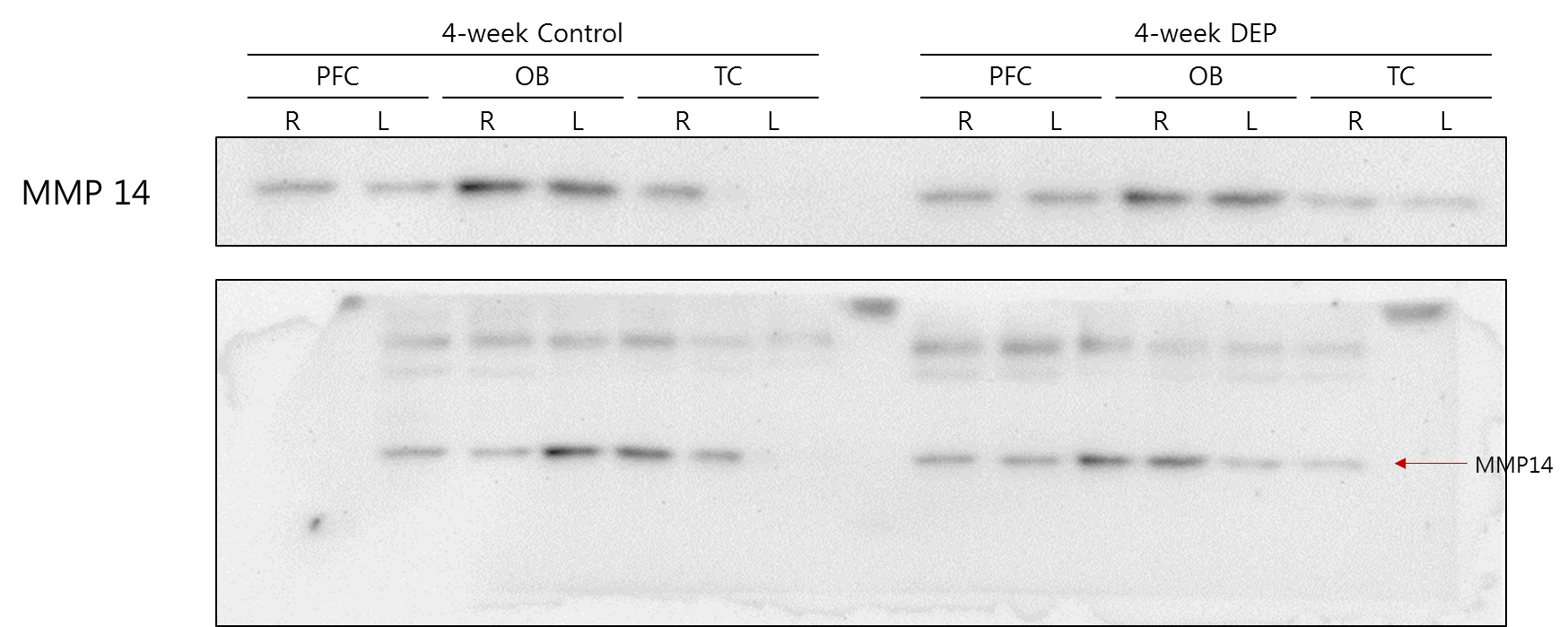


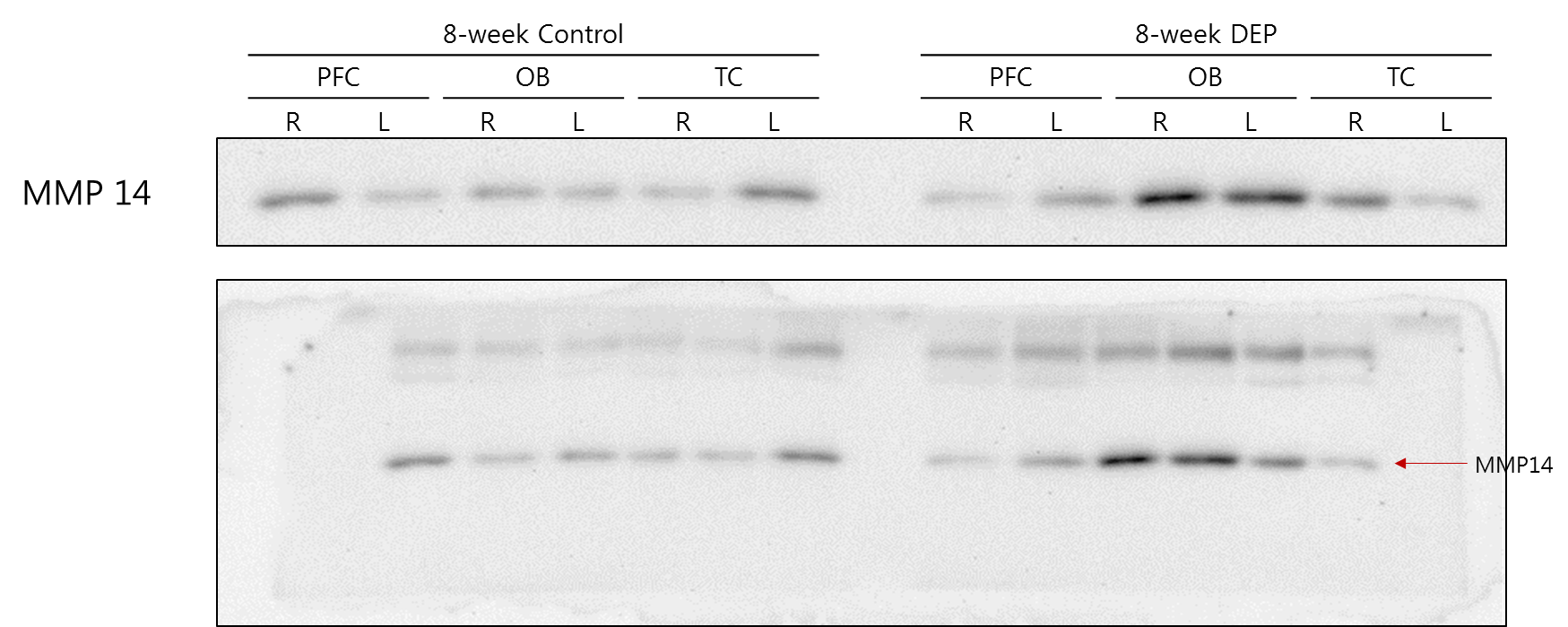

Supplement: Supplementary file 1 — Supplement Table [file 41598_2019_55956_MOESM1_ESM.docx]
